# Simultaneous enhancement of electrical conductivity and interlaminar fracture toughness of carbon fiber/epoxy composites using plasma-treated conductive thermoplastic film interleaves†

**DOI:** 10.1039/c8ra05366a

**Published:** 2018-07-30

**Authors:** Wei Li, Dong Xiang, Lei Wang, Eileen Harkin-Jones, Chunxia Zhao, Bin Wang, Yuntao Li

**Affiliations:** School of Materials Science and Engineering, Southwest Petroleum University Chengdu 610500 China dxiang01@hotmail.com yuntaoli@swpu.edu.cn; School of Engineering, University of Ulster Jordanstown BT37 0QB UK

## Abstract

Multiwalled carbon nanotube (MWCNT)-doped polyamide 12 (PA12) films with various nanofiller loadings were prepared *via* a solution casting method to simultaneously improve the electrical conductivity and fracture toughness of carbon fiber/epoxy (CF/EP) composites. The films were interleaved between CF/EP prepreg layers and melted to bond with the matrix during the curing process. To improve the interfacial compatibility and adhesion between the conductive thermoplastic films (CTFs) and the epoxy matrix, the CTFs were perforated and then subjected to a low temperature oxygen plasma treatment before interleaving. Fourier transform infrared (FTIR) spectra results confirm that oxygen-containing functional groups were introduced on the surface of the CTFs, and experimental results demonstrate that the electrical conductivity of the laminates was significantly improved. There was a 2-fold increase in the transverse direction electrical conductivity of the laminate with 0.7 wt% MWCNT loading and a 21-fold increase in the through-thickness direction. Double cantilever beam (DCB) tests demonstrated that the Mode-I fracture toughness (*G*_IC_) and resistance (*G*_IR_) of the same laminates significantly increased by 59% and 113%, respectively. Enhancements of both interlaminar fracture toughness and electrical conductivity are mainly attributed to the strong interfacial adhesion achieved after plasma treatment and to the bridging effect of the carbon nanotubes.

## Abstract 2

The electrical conductivity and interlaminar fracture toughness of carbon fiber/epoxy composites are simultaneously enhanced by plasma-treated MWCNT-doped PA12 film interleaves. This work is important for the LSP and EMI properties of CFRP.

## Graphical abstract

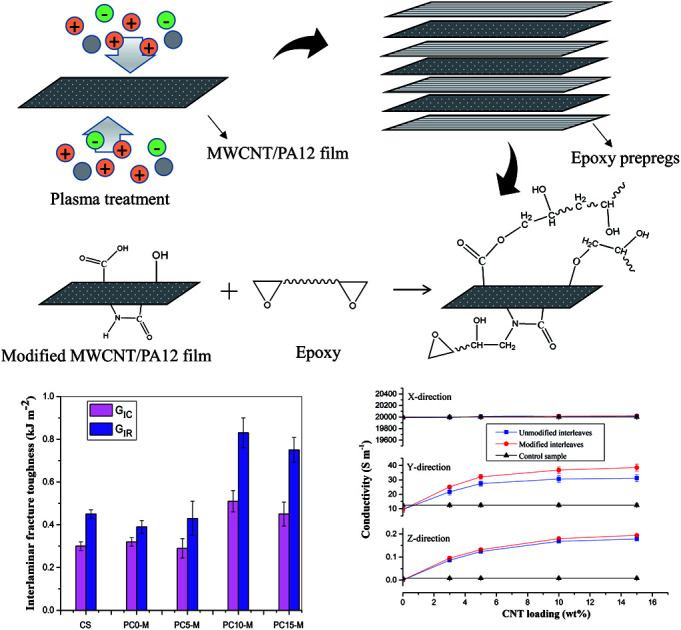

## 1. Introduction

In recent years, there has been much interest in developing composite materials for large primary structures on military and civil aircraft in order to achieve lighter structures for significantly reduced fuel consumption and reduced environmental impact. Compared to conventional metals such as aluminum and steel, carbon fiber reinforced epoxy (CF/EP) composites have higher specific strength and modulus, fatigue strength, excellent environmental stability, and design flexibility. Therefore, there are increasing demands for CF/EP composites in the aerospace field.^1^ It has been reported that the amount of composite material used in a Boeing 787 Dreamliner and Airbus A350 XWB accounts for more than 50% of the aircraft's structural weight, and this greatly reduces fuel consumption (by 20%) as well as production and maintenance costs.^2–4^

Electrical conductivity is an important parameter for composites used in the aerospace field. Although carbon fiber has a high conductivity, epoxy resin is an electrically insulating material and the electrical conductivity of carbon fiber composites does not meet the requirements of lightning protection (LSP) and electromagnetic interference (EMI) shielding for applications in aerospace.^5,6^ The conventional method used to increase conductivity of composite materials is to bond aluminum or copper mesh to the surface of the structure; however, using this increases the total weight of the system, the process is relatively complicated, and there is low maintenance efficiency. In recent years, there has been an increasing interest in developing conductive composites *via* the addition of carbon or metal nanofiller to form three-dimensional conductive network structures with particles such as carbon nanotubes (CNTs),^7–10^ graphene nanoplatelets,^11^ carbon black,^5^ or silver nanowires.^12^ In particular, CNTs exhibit extraordinary electrical conductivity and have mechanical properties that make them one of the most suitable conductive fillers for preparing conductive polymer composites.^13–16^ However, the high aspect ratio and strong van der Waals interactions between nanoparticles cause severe aggregation and poor dispersion, which may lead to adverse consequences. Oxidation can improve the dispersibility of carbon nanotubes but using strong acids as oxidants can impair mechanical and electrical properties, and this process can be dangerous.^7,17^

Composite structures are also susceptible to damage from accidental impact or cyclic loading (tension, compression, shear, bending, *etc.*) during service, storage, and routine maintenance.^18,19^ This damage is mainly in the form of delamination failure and matrix microcracking. In particular, invisible delamination may occur in the interlayer resin-rich region, which results in a drastic drop in the load-carrying capacity of the composite structure;^20,21^ this is the key failure mode limiting the service life of a composite.

The fracture toughness of carbon fiber/epoxy (CF/EP) composites is determined by the toughness of the matrix and the interfacial strength between the reinforcement and the matrix.^22,23^ In recent years, thermoplastic polymers in the forms of particles, films, nanofiber veils,^19,24,25^ or nanofillers^5,26–28^ have been introduced as interleaves in the damage-prone interlaminar regions to improve interlaminar fracture toughness. Various effective mechanisms, such as ductile deformation, void formation and crazing, and bridging and pull-out have been identified as enhancing the energy absorption capability of the matrix and inhibiting generation and growth of cracks between layers.^29–31^ These methods improve the composite material damage tolerance without affecting the mechanical and thermal properties of the resin matrix itself and are compatible with existing manufacturing processes. Arnold *et al.*^31^ inserted different physical forms of PA12 as an interlaminar layer and results showed a clear improvement in Mode I and II fracture toughness. White *et al.*^32^ also reported improved CFRP delamination toughness using partially cured epoxy thin films containing PA12 particles and multi-walled carbon nanotubes (MWCNTs). PA is an excellent thermoplastic engineering plastic with high fracture toughness and fatigue resistance and incorporating it into an epoxy matrix can increase the ability of the matrix to absorb fracture energy.

Interfacial compatibility and adhesion between the introduced interleaf and the matrix governs the efficiency of stress transfer and is an important factor for the mechanical performance of the composite.^23,33,34^ It is possible for a very strong interphase interaction to produce high stress transfer efficiency and to withstand large fracture loads, whereas a poor interface can lead to debonding of the interface and promote crack initiation. Various methods have been developed to introduce functional groups that can participate in the epoxy curing reaction to improve interfacial interactions through covalent bonds or noncovalent interactions. A chemical method is to introduce functional groups through chemical synthesis reactions on a ductile toughening agent, such as reactive liquid rubber.^35,36^ However, this method usually decreases the glass transition temperature (*T*_g_) and the strength and elastic modulus of the epoxy resin. Moreover, the synthesis process is complicated and harmful. In recent years, there has been increased research activity aimed at improving interface strength using low-temperature plasma treatment with the introduction of various functional groups. Low-temperature plasma treatment causes less damage to the inherent properties of a material and increases interfacial interactions by improving surface activity.^37–39^ Compared with chemical methods, low-temperature plasma treatment is simpler, cleaner and scalable.

In this work, we prepared MWCNT doped conductive thermoplastic (PA12) films (CTFs) using a solution casting method. Perforations were made in the films to facilitate resin flow during resin infusion. The CTFs were oxygen plasma treated to improve the interfacial interaction with the epoxy matrix and then interleaved between prepreg layers in order to simultaneously improve the interlaminar fracture toughness and conductivity of the CF/EP composites. To the best of our knowledge, the levels of enhancement achieved simultaneously for both electrical conductivity and fracture toughness of the CF/EP composite have not been reported elsewhere for such low loadings of MWCNTs in the final laminate.

## 2. Materials and methods

### 2.1. Materials

MWCNTs (NANOCYL NC7000) were obtained from Nanocyl S.A. (Sambreville, Belgium). The average diameter and length of the MWCNTs were 9.5 nm and 1.5 μm, respectively. The density of the MWCNTs was 2.0 g cm^−3^. High temperature CF/EP unidirectional prepregs were purchased from BONATECH Advanced Materials Company (Beijing, China), and the physical and mechanical properties of the constituent materials are summarized in Table 1. PA12 powder (VESTOSINT® 2161, density of 1.02 g cm^−3^ and melting temperature of 184 °C) was supplied by Evonik Industries AG (Essen, Germany). *N*,*N*-Dimethylformamide (DMF) was sourced from Kelong Chemical Reagent Company (Chengdu, China).

**Table 1:** Specifications of unidirectional CF/EP prepregs

Parameter	Specification
Carbon fiber model	T700
Fiber mass per unit area (g m^−2^)	132 ± 5
Resin content (wt%)	35 ± 3
Monolayer prepreg thickness (mm)	0.12
Epoxy resin model	B-228H
*T*_g_ of epoxy resin (°C)	260
Tensile strength in fiber direction (MPa)	2500
Bending strength (MPa)	1650

### 2.2. Fabrication of plasma treated porous CTFs

CTFs with different MWCNT contents (0, 0.2, 0.5, 1, 3, 5, 10, and 15 wt%) were manufactured using a solution casting method. Briefly, MWCNTs were first added to DMF, stirred for 15 min, and sonicated for 1 h. PA12 powder was then added to the MWCNT/DMF suspension, and the mixture was vigorously stirred at 160 °C for 3 h. The resulting MWCNT/PA12/DMF mixture was poured into a glass mold (150 mm × 150 mm × 8 mm) and dried at 80 °C for 12 h. The CTFs had a thickness of about 15 μm and were dried in a vacuum oven at 80 °C for 3 h to remove residual solvent. Finally, the CTFs were perforated with 1 mm diameter holes using a customized needle plate. The density of holes was 25 holes per square centimeter.

A low-temperature plasma processor (OMEGA-DT03, China) was used to treat the films. For the plasma treatment process, oxygen was at a gas flow rate of 40 sccm with a plasma power of 120 W and exposure time was 6 min.

### 2.3. Fabrication of CF/EP composite laminates and test specimens

**Fig. 1:**
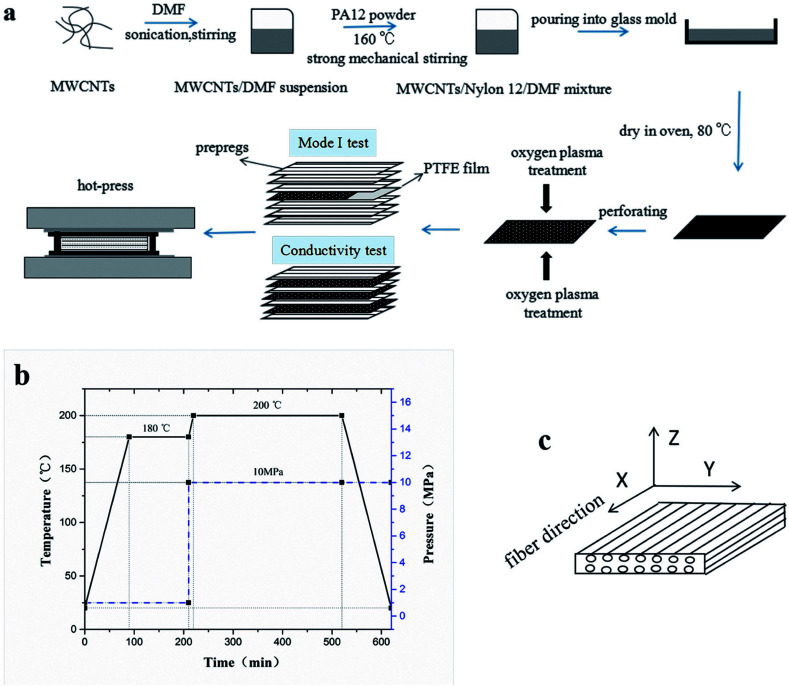
Fabrication of CTF-interleaved CF/EP composite laminates. (a) Schematic diagram of the fabrication process, (b) prepreg curing process, and (c) identification of the three directions of the laminate.

To prepare the composite laminates, unidirectional CF/EP prepregs were cut into pieces of dimension 150 mm × 150 mm. The lay-up was [0°]_12_ for the conductivity tests, and modified or unmodified CTFs were interleaved between every two adjacent prepregs (eleven CTFs and twelve prepreg layers in total), where the top and bottom layers were prepregs. The lay-up of laminates for Mode I interlaminar fracture toughness tests was [0°]_24_. Modified or unmodified porous CTF was interleaved between the twelfth and thirteenth prepreg layers. In addition, a PTFE film that was 13 μm thick was coated with a release agent on both surfaces and was implanted in close proximity to the CTF to create a starter crack of length 50 mm. A schematic diagram of the fabrication process of CTF interleaved CF/EP composite laminates is shown in Fig. 1a.

After the layup, the preform was cured using a hot-press to manufacture the laminates in accordance with the curing process provided by the supplier and shown in Fig. 1b. A release agent was sprayed on the surface of the steel mold so that the cured laminates could be easily demolded. Finally, the composite laminate was cooled to room temperature under 10 MPa of pressure. For the interlaminar fracture toughness tests, the average thickness of the samples was about 4 mm. For the conductivity test, the average thickness of the specimens was about 2 mm with and without conductive film. Inserting the multilayer film into the laminate does not significantly increase the thickness of the laminate. The fiber volume fraction (VF) was calculated using1
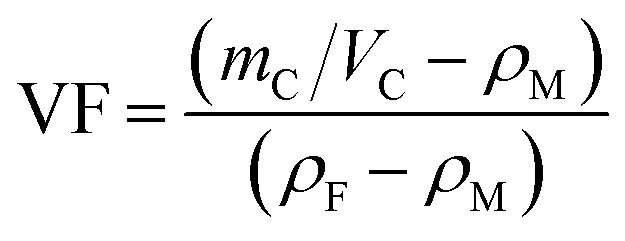
where *m*_C_ and *V*_C_ represent the mass and volume of composite, respectively; *ρ*_M_ and *ρ*_F_ are the density of the matrix and fiber, respectively. A CF/EP laminate without interleaves was also prepared and labeled as a control sample (CS). Test specimens were cut from the cured laminates using a water jet cutter. Conductivity test samples had dimensions of 10 mm × 10 mm × 2 mm. Fig. 1c shows the identification of the three directions of the laminate. For the interlaminar fracture toughness test, the specimens had dimensions of 140 mm × 20 mm × 4 mm. Note that before the laminate was cut, the end of the PTFE film was exactly located and marked. Variations in the width and thickness of all of the test specimens did not exceed 0.5 mm and 5% of the mean value, respectively. Table 2 shows a summary of the different composite laminate samples.

**Table 2:** Summary of the different composite laminate sample codes

Sample code		Interleaf	Fiber volume fraction (vol%)	MWCNTs mass fraction (wt%)
Without plasma treatment	Plasma treatment			
CS	—	—	63 ± 5	—
PC0	PC0-M	0 wt% MWCNTs/PA12	62 ± 5	—
PC5	PC5-M	5 wt% MWCNTs/PA12	62 ± 5	0.35
PC10	PC10-M	10 wt% MWCNTs/PA12	62 ± 5	0.7
PC15	PC15-M	15 wt% MWCNTs/PA12	62 ± 5	1.05

### 2.4. Characterization

#### 2.4.1 Ultraviolet-visible (UV-vis) spectroscopy

Dispersion quality and stability of the MWCNT/DMF suspension was demonstrated using UV-vis spectroscopy (UV-2600, Shimadzu, Japan). This was used to measure light absorption of the suspension to quantify individual MWCNTs or dispersion properties of their agglomerates.^40,41^

#### 2.4.2 Microscopy

Conductive networks below the CTF surface and the fracture surface morphology of DCB samples were observed using a JSM 7500F field emission scanning electron microscope (FE-SEM) under an accelerating voltage 20 kV. The fracture surface of composite laminates was sputtered with gold before observation. To make the conductive network under the polymer matrix visible by emitting enriched secondary electrons after charging, CTFs were not sputtered with gold. Part of the PA phase was chemically etched with DMF under ultrasound before cross-section observation was made of the conductivity test specimens.

#### 2.4.3 FTIR spectroscopy

Surface chemical analysis of films after plasma treatment were characterized using FTIR (Nicolet 6700, Thermo Scientific, USA). The FTIR test was performed immediately after the plasma treatment.

#### 2.4.4 Conductivity testing

Volume conductivities of CTFs were measured at ambient temperature using a two-point probe method picoammeter (Keithley 6485) and a DC voltage source (Tektronix PWS4323) at a constant voltage of 1 V. The CTF film were cut into strip-shaped samples with dimensions of 50 mm × 10 mm, and the distance between the electrodes was 30 mm. Conductivities of the prepared CF/EP laminates were measured at ambient temperature using a four-wire method with a KEITHLEY 2000 multimeter. Conductivities of the composite laminate samples with a dimensions of 10 mm × 10 mm × 2 mm were also measured. Because of the anisotropy of the fibers, conductivity in three directions (fiber, transverse, and through-thickness directions) was measured. All conductivity tests were performed on at least three specimens. Specimen surfaces were first polished with abrasive paper, cleaned with acetone, and then dried. Finally, the test surface was coated with a layer of conductive silver paste to minimize contact resistance. Conductivities (*σ*) of the CTFs and CF/EP laminates were calculated according to eqn (2)2
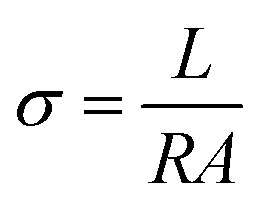
here, *A* is the cross-sectional area, *R* is the volume resistance, and *L* is the length of the specimen. *A* and *L* represent different geometrical dimensions of the specimen for the three-direction measurements.

#### 2.4.5 Mode I interlaminar fracture toughness test

**Fig. 2:**
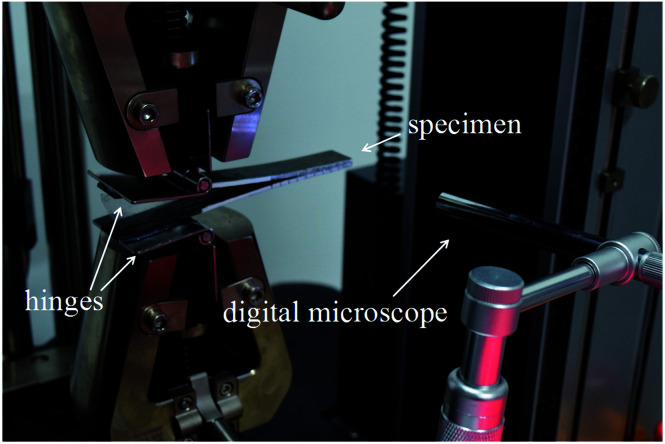
Mode I test set-up with a digital microscope.

Mode I interlaminar fracture toughness of the laminate was measured using double-cantilever-beams (DCB) at ambient conditions on a universal testing machine (SANS CMT4104) according to ASTM D5528. Two hinges were adhered to the pre-cracked end of the specimen. At least five specimens were tested, and, to visually detect the onset and propagation of cracks, the edges of each specimen were coated with a thin layer of water-based white paint and marked with thin vertical lines every 1 mm. A digital microscope with a maximum magnification of 500 was positioned to observe crack growth at the delamination front. Fig. 2 shows the set-up of the Mode I test. Total crack length (*a*) is the sum of the distance from the loading line to the end of the inserted PTFE film plus the increment of growth, which is determined from the tick marks. Tests were conducted at a speed of 1 mm min^−1^ in the displacement control mode. Mode I interlaminar fracture toughness was calculated according to the modified compliance calibration (MCC) method, as expressed in eqn (3)3
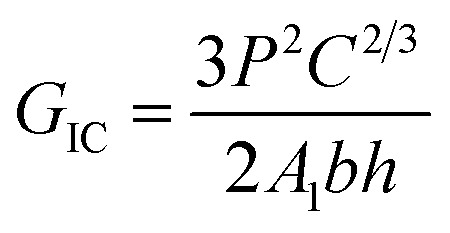
where *P* is the load corresponding to the defined crack length (*a*). Compliance (*C*) is the ratio of the load point displacement to the load that corresponds to a defined crack length. *A*_1_ is the slope of the line on the least-squares plot of the crack length normalized by specimen thickness (*a*/*h*) as a function of the cube root of compliance (*C*^1/3^). *b* and *h* are the width and thickness, respectively, of the specimen. The calculated value of *G*_IC_ increased 5% in compliance with the original linear region of the force–displacement curve.

## 3. Results and discussion

### 3.1. Conductivity of CTFs

**Fig. 3:**
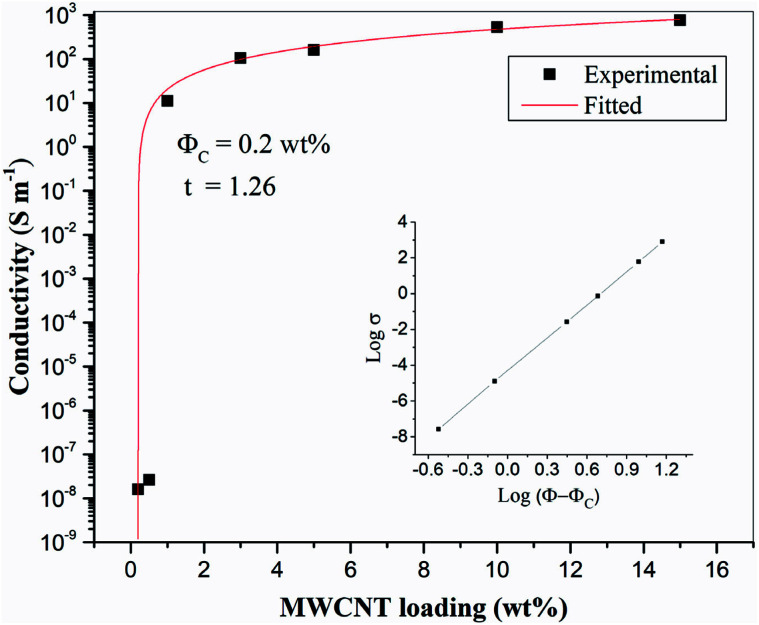
Electrical conductivity of CTFs with various loadings of MWCNTs (inset: log–log plot of the conductivity *versus Φ* − *Φ*_C_).

Firstly, the electrical properties of the CTFs were investigated. Fig. 3 shows the experimental data and the fitted curve of CTF conductivities as a function of MWCNT loading. Pure PA12 film is completely insulating and conductivity gradually increases with the addition of CNTs, which exhibit a clear percolation behavior. At 1 wt% MWCNT loading, the electrical conductivity increases sharply from 10^−8^ S m^−1^ to 11.2 S m^−1^, which is an increase of eight orders of magnitude. Conductivity of the CTFs with 5 wt%, 10 wt%, and 15 wt% loadings are 162.6 S m^−1^, 533.3 S m^−1^, and 775.2 S m^−1^, respectively. According to classical statistical percolation theory,^42^ the percolation threshold for the CTFs can be calculated. The relationship is given by the scaling law shown in eqn (4)4
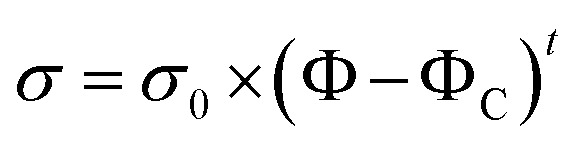
where *σ* is the conductivity of the system, *σ*_0_ is the conductivity of the nanofiller, *Φ* is the mass fraction of the fillers, *Φ*_C_ is the filler concentration at the percolation threshold, and *t* is the critical exponent; the critical exponent depends on the dimensionality of the conductivity network. The values of *σ* are approximately 1.6–2 in a 3D system and 1–1.3 in a 2D system. *Φ*_C_ and *t* were determined *via* a least square fitting of the experimental data; *Φ* is an independent variable, and *σ* is a dependent variable. The inset of Fig. 3 shows the experimental values of conductivity plotted as log *σ versus* log(*Φ* − *Φ*_C_), and the values of *Φ*_C_ and *t* were varied until the best linear fit with minimum error was obtained for the plot. The fitting results show that the CTF composite system has a low critical concentration (*Φ*_C_ = 0.2 wt%). The critical exponent (*t*) is 1.26, and this low critical exponent indicates that a complete conductive path was formed in the film and generally follows a 2D model because of restricted electron hopping between MWCNTs in the transverse direction.

**Fig. 4:**
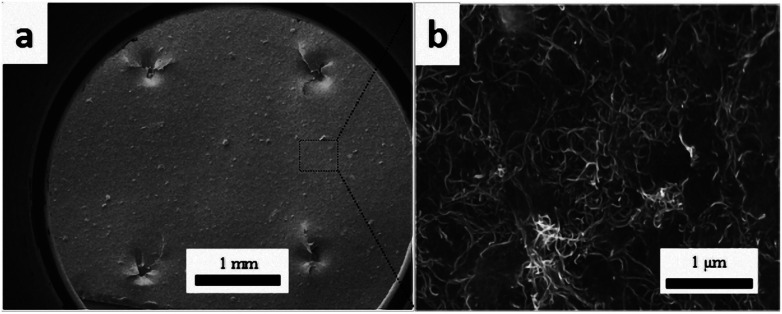
SEM images of CTF with 10 wt% MWCNTs loading: (a) at low magnification and (b) local high magnification image.

Fig. 4 shows SEM images of the 10 wt% MWCNTs film after perforation. Holes (each about 1 mm in diameter) are arranged in an orderly manner with spacing of about 2 mm. The high porosity enables epoxy penetration through the laminate structure. As can be seen in higher magnification images, the CNTs embedded in the matrix are curved and wavy (due to high loading). Several authors have found that waviness has a negative effect on percolation, the percolation threshold having been found to increase with increasing CNT waviness.^43–46^ A large number of interlaced, individual MWCNTs and loose aggregates are uniformly distributed in the matrix and form a dendritic conductive network structure. Researchers have reported that a dendritic network formed by uniformly dispersed MWCNT aggregates may be more conducive to electron transfer.^47,48^ Therefore, it may be more effective to increase the conductivity of the CF/EP composite laminates by introducing uniformly dispersed micro-sized MWCNT aggregate CTF between the layers. Fig. S1† shows the UV-vis spectra of the prepared CNT suspensions (15 wt%); the spectra were recorded after the suspensions were stored for different lengths of time (one hour, one week, and one month). There is no significant change in peak absorption (263 nm). Over time, there is only a small change in peak absorption, and there is no obvious sediment after one month, indicating that the suspension was stable.

### 3.2. Electrical conductivity of CF/EP composite laminates

**Fig. 5:**
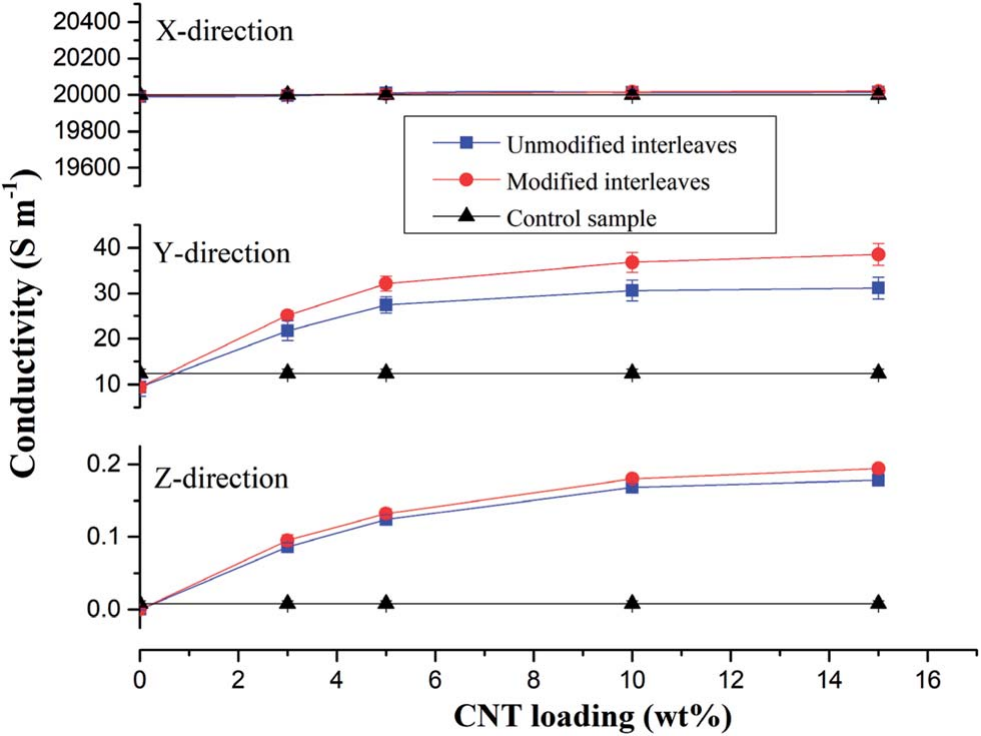
Conductivities of composite samples and the control sample. Conductivities in the fiber direction (*X*-direction), transverse direction (*Y*-direction), and through-thickness direction (*Z*-direction) of the composite interleaved with unmodified and modified interleaves with various MWCNT loading.

Control samples and composite laminates interleaved with unmodified and modified CTFs with different MWCNT loadings were prepared at the same conditions for this conductivity study. Fig. 5 shows average values of volume electrical conductivity changes in the fiber direction (*X*-direction), transverse direction (*Y*-direction), and through-thickness direction (*Z*-direction). We can observe from the experimental results there were no significant changes in conductivity along the fiber direction (*X*-direction) for all of the test samples. The values are all about 20 000 S m^−1^; indicating, as expected, that the interleaving does not affect the electrical properties in the fiber direction. The laminate interleaved with a pure PA film is nonconductive in the *Z*-direction; the volume electrical conductivity is lower than 10^−6^ S m^−1^, and the conductivity in the *Y*-direction also decreases. These observations are attributed to the insulating PA film hindering fiber contact in the thickness direction so that a conductive path is not formed.^6^ However, for the samples interleaved with CTFs, conductivities in the *Y* and *Z*-directions both increase as the MWCNT loading increases in the film. The trend then plateaus when the weight fraction of CNTs in the film reaches 5 wt%. The increase is obviously attributed to the excellent electrical conductivity of the MWCNTs and to reduced electrical resistance of the interlaminar resin-rich region. When the mass fraction of MWCNTs in the film is greater than 10%, the electrical conductivity of the composite laminate does not increase significantly. As can be seen from Fig. 5, the conductivity of the laminate interleaved with the plasma treated CTFs shows a slight improvement compared to that of the laminate interleaved with unmodified CTFs. Conductivities of the composite laminate PC10-M in the *Y*- and *Z*-directions are 36.8 S m^−1^ and 0.18 S m^−1^, respectively, which are 2-fold and 21-fold improvements compared to the control sample. In this case, the weight fraction of MWCNTs is equal to only 0.7 wt% of the entire composite laminate. Compared with the experimental results reported in the literature, the improved electrical conductivity observed in our investigation is significant. For example, in a previous study SWCNTs were sprayed onto the surface of prepregs to prepare a laminate, and the conductivity in the thickness direction improved by 144% for 2 wt% SWCNT compared to without SWCNTs.^49^ The electrical conductivity of a CF/EP composite laminate that incorporated copper chloride and 3 wt% carbon black increased by 54% and 45% in the transverse and through-thickness directions, respectively.^5^ Out-of-plane electrical conductivity of a laminate with 2 vol% GNPs improved by more than 200% compared with that of a laminate without GNPs.^50^ Another study reported that carbon fiber-based laminates that included a 0.1 wt% MWCNT-doped resin led to a 30% increase in through-thickness electrical conductivity.^51^ The conductivity enhancements achieved in this study are important and bring the composite into the lightning strike protection (LSP) and electromagnetic interference (EMI) shielding range of conductivities.

**Fig. 6:**
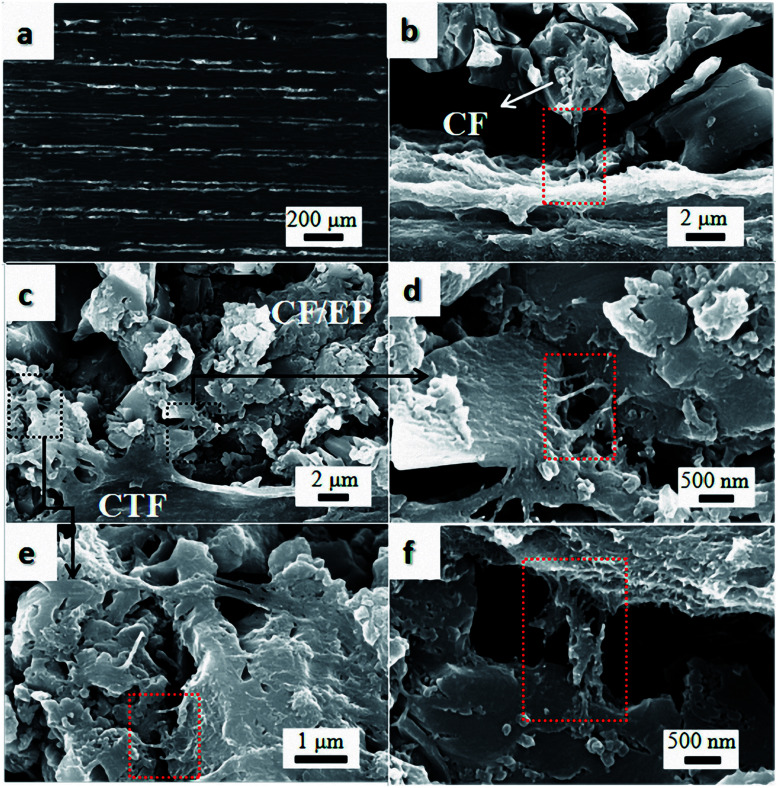
SEM images of DMF etched cross-section of PC10-M. (a) The surface was not sputtered with gold, and (b–f) conductive networks formed *via* MWCNTs between the CTF and CF/EP.

Fig. 6 shows SEM images of the cross-section of the interleaved sample PC10-M. The cross-section of the nonsputtered sample is shown in Fig. 6a. The periodic stack structure is obvious, and the bright zone represents the CTF interlayers, which indicate lower resistivity than the dark zone.^12^Fig. 6b–f show a close observation of the cross-section of the interleaved specimen with plasma-treated 10 wt% CTF. Part of the PA phase was chemically etched with DMF under ultrasound before SEM observation, and this shows that a more interconnected conductive network formed through a bridging of the carbon nanotubes between CTF and CF/EP and between the carbon fibers. These observations can be clearly seen in local magnified images (Fig. 6d–f). Therefore, improved conductivity is mainly attributed to the presence of a large number of conductive networks that reduce the electrical resistance of the resin-rich region.

### 3.3. Mode I interlaminar fracture toughness

**Fig. 7:**
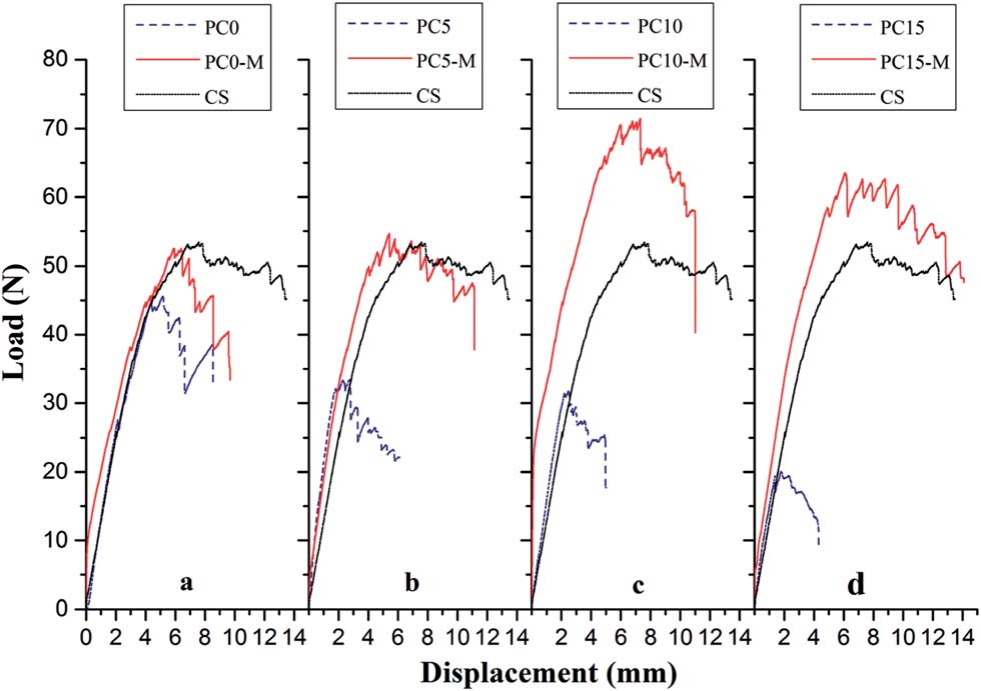
Comparison of typical DCB load–displacement curves for composites with different interlayers: (a) CS, PC0, and PC0-M, (b) PC5 and PC5-M, (c) PC10 and PC10-M, and (d) PC15 and PC15-M.

Interlaminar fracture toughness of composite laminates interleaved with the CTFs that had MWCNT loadings of 0, 5, 10, and 15 wt% was also investigated. Fig. 7 shows typical load-crack opening displacement (COD) curves obtained using average values from DCB tests of five specimens for each type of laminate interleaved with unmodified (PC0, PC5, PC10, and PC15) and modified interlayer films (PC0-M, PC5-M, PC10-M, and PC15-M) and for CS of laminates. As can be observed in Fig. 7, the load increases rapidly in the initial loading stage. The critical loads at the peak of the curve for the unmodified interleaved samples show a decrease compared to control samples, and the load drops rapidly after the peak. With an increase in the MWCNT loading in the film, the decrease is more significant, and a shorter crack opening displacement is observed. These indicate a rapid failure mode during loading. However, critical loads of the modified CTF-interleaved laminates (PC5-M, PC10-M, and PC15-M) are higher than those of the CS and are maintained at a high load plateau. However, there is a slight decrease in the critical load of sample PC0-M compared to that of the CS. The load–displacement curves of modified CTF-interleaved laminates fluctuate more obviously with crack growth. This indicates a ductile fracture mode behavior. As the MWCNT content increases in the film, the highest loads occur in the case of PC10-M, which is about 44% higher than that of the CS. However, as the MWCNT loading increases further (PC15-M), there is a slight decrease in the critical load compared to that of PC10-M.

**Fig. 8:**
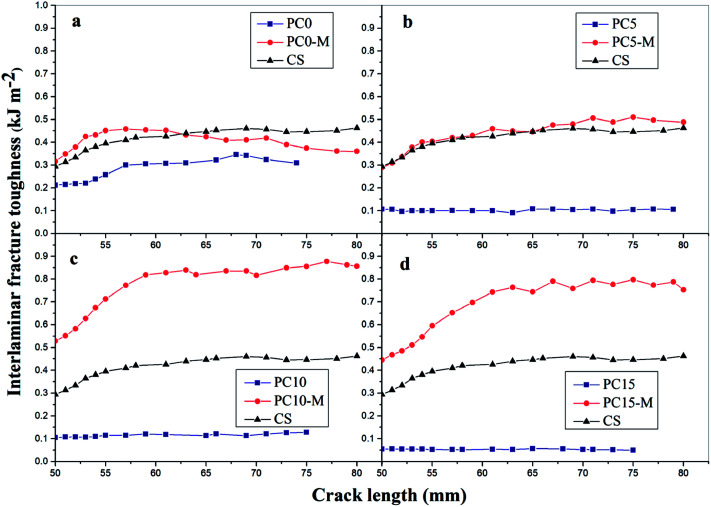
Mode-I fracture toughness-crack length curves for composites with different interlayers: (a) CS, PC0, and PC0-M, (b) PC5 and PC5-M, (c) PC10 and PC10-M, and (d) PC15 and PC15-M.

**Fig. 9:**
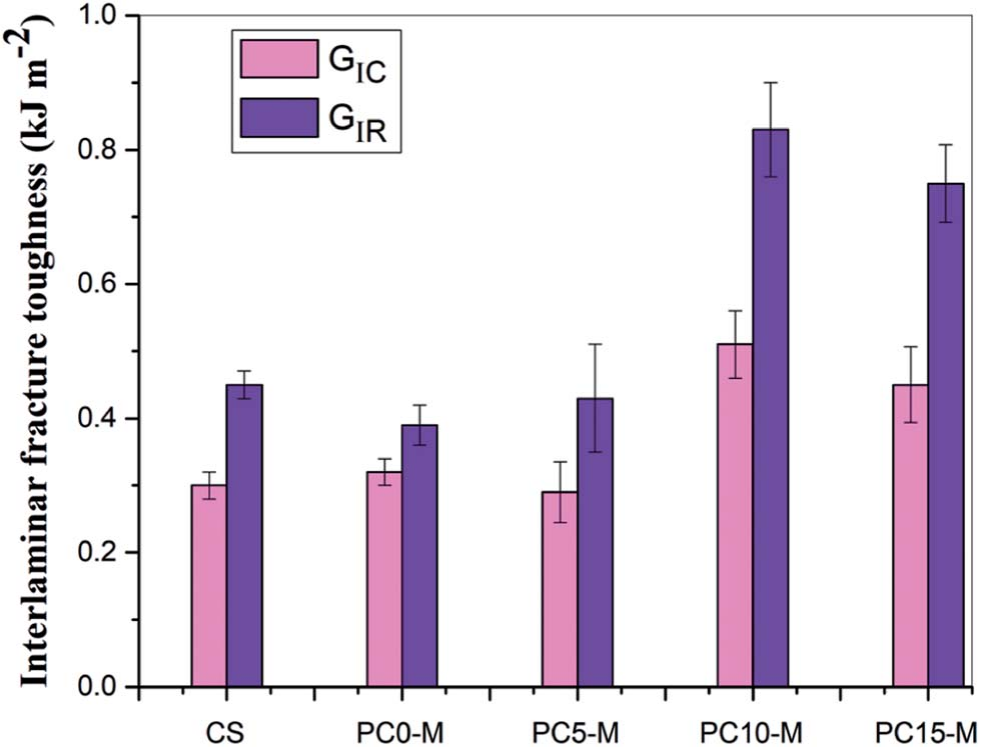
Comparison of Mode-I fracture toughness and resistance for composites interleaved with modified films with various MWCNT loadings.

Fig. 8 shows comparisons of calculated mode I interlaminar fracture toughness as a function of crack length (*R*-curves). Fig. 9 shows the corresponding values of *G*_IC_ and *G*_IR_ with added error bars to represent Mode-I initial fracture toughness and fracture resistance, respectively. The value of *G*_IC_ was obtained from the initial point of cracking for *R*-curves (50 mm), and the value of *G*_IR_ is the average value of fracture toughness within the range of crack lengths from 60 mm to 80 mm in the *R*-curves. The results show that the trend in *R*-curves is similar to the load–displacement curve (Fig. 7). The unmodified film interlayer had a negative effect on the interlaminar fracture toughness, and this effect increases with an increase in CNT loading. Fracture toughness is significantly lower than that of the control sample and was almost invariant with crack propagation. This may be mainly attributed to the weak interfacial interaction between the CTF and CF/EP. This test result for the unmodified interleaved samples is similar to the results reported in other research that also used PA12 modified with nanoparticles as the interleaf material.^52^ In this work, although the fracture toughness values of sample PC0-M did not show a significant improvement, the test results of laminates interleaved with modified CTFs have significantly improved fracture toughness values compared with those of the control samples and unmodified film counterparts. This indicates the toughening effect of the CNTs. Fig. 9 clearly shows a slight increase in the *G*_IC_ value of the PC0-M laminate, whereas the fracture resistance (*G*_IR_) slightly decreases compared with that of the control sample. As the carbon nanotube content in the film increase, both *G*_IC_ and *G*_IR_ increase up to maximum value for the PC10-M sample. The maximum values of *G*_IC_ and *G*_IR_ are 0.51 kJ m^−2^ and 0.83 kJ m^−2^, respectively, equivalent to increases of 59.3% and 112.8% compared to the control sample values of 0.3 kJ m^−2^ and 0.45 kJ m^−2^, respectively. The values of *G*_IC_ and *G*_IR_ then decrease slightly with a further increase to 15 wt% of added MWCNTs. This may be due the presence of agglomerates which would provide stress concentration sites.

### 3.4. Fracture toughness mechanism

#### 3.4.1 FTIR analysis

**Fig. 10:**
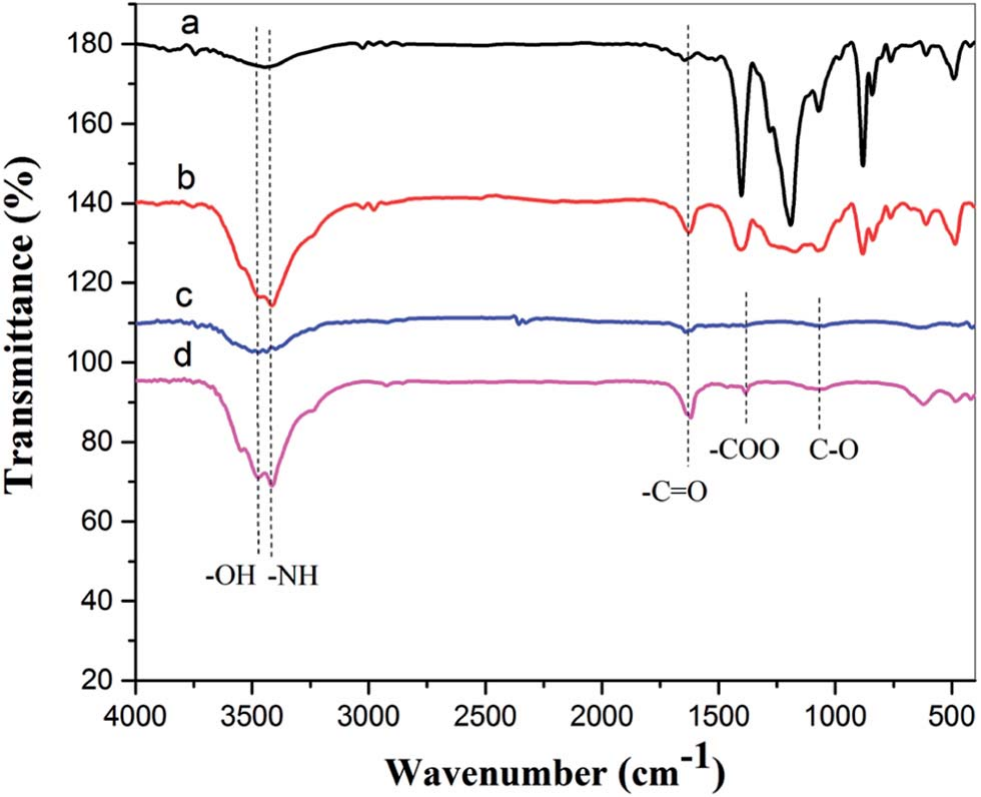
FTIR spectra of (a) pure PA12 film, (b) oxygen plasma-treated pure PA12 film, (c) CTF with 10 wt% MWCNT loading, and (d) oxygen plasma-treated CTF with 10 wt% MWCNT loading.

FTIR spectra for pure PA12, oxygen plasma-treated pure PA12 film, CTF with 10 wt% MWCNT loading, and oxygen plasma-treated CTF with 10 wt% MWCNT loadings are shown in Fig. 10. The peak at 3480 cm^−1^ corresponds to the O–H stretching vibration and was detected in spectra of plasma-treated pure and doped films. The peak at 3415 cm^−1^ corresponds to the N–H stretching vibration and is observed in all four spectra; this is attributed to the PA amide group (–CONH–). However, the shape of the peak at 3415 cm^−1^ is sharper in the plasma-treated sample. This may be because of residual nitrogen in the discharge chamber during plasma processing. Similarly, the peak at 1640 cm^−1^ corresponds to the C=O stretching vibration, and this peak is sharper after oxygen plasma treatment. The spectra of the oxygen plasma-treated doped film exhibit other peaks at 1380 cm^−1^ and 1070 cm^−1^, and these may correspond to the –COO symmetrical stretching vibration and the C–O stretching vibration.^38^

**Fig. 11:**
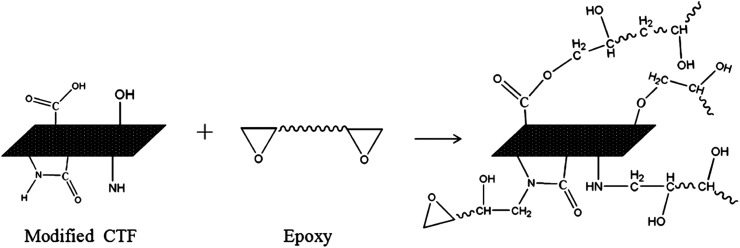
Possible interfacial reactions between modified films and the epoxy matrix.

The FTIR results demonstrate that reactive functional groups have been incorporated onto the surfaces of both the unfilled PA12 film and CTF after oxygen plasma treatment. Increased functional groups enhance the surface compatibility and reactivity of CTF so that it can chemically bond to the epoxy matrix, which leads to improved interfacial compatibility.^53,54^ Chemical bonding at the interface seems to be closely related to ring-opening reactions between epoxide groups of the epoxy matrix and active hydrogen-containing functional groups of the modified CTF. Fig. 11 shows possible ring-opening reactions between the epoxy and introduced active hydrogen-containing functional groups on the surface of the CTF. Creation of a chemically bonded interface is likely to be of great benefit for stress transfer in the interlaminar region of CF/EP composites.

#### 3.4.2 Fracture morphology

**Fig. 12:**
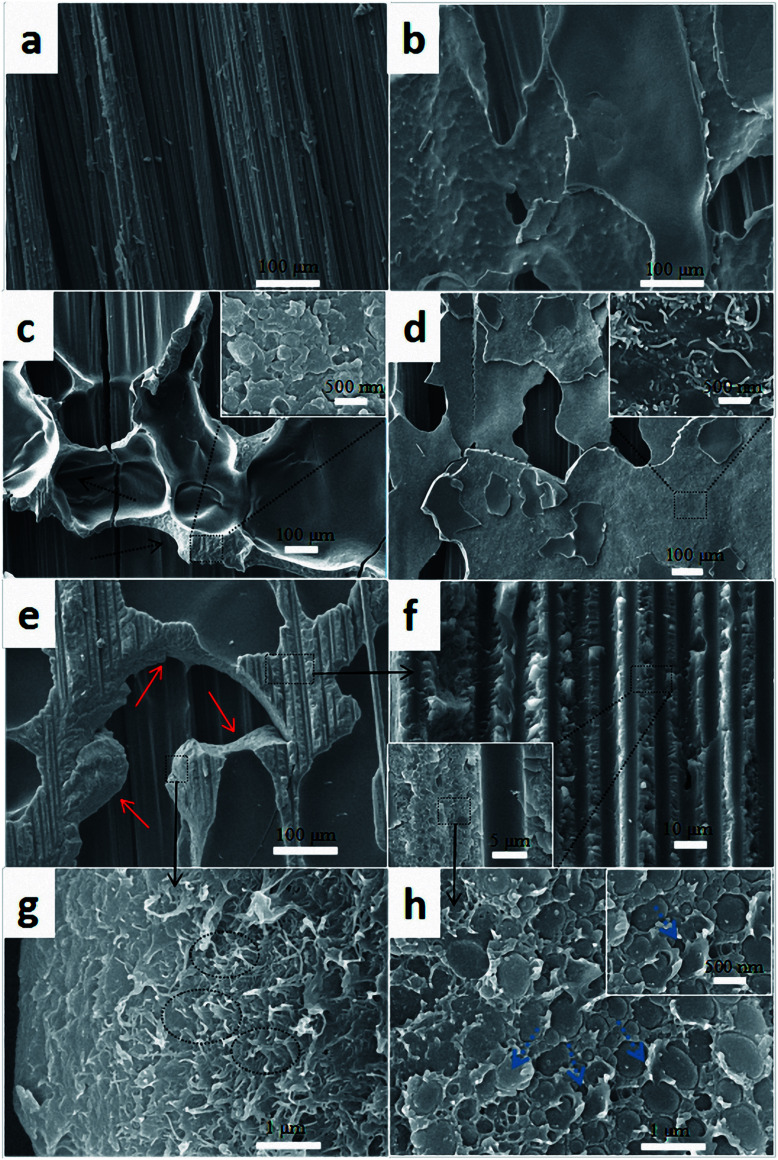
Comparison of SEM images of fracture surfaces of the composites with different interlayers: (a) CS, (b) PC0, (c) PC0-M, (d) PC10, and (e–h) PC10-M.

Fracture surfaces were carefully analyzed to explore the enhancement mechanism of CTF after plasma treatment. Fig. 12 shows fracture surface morphologies of composite laminates after the DCB test. As shown in Fig. 12a, the fracture surface of the control sample laminates exhibits a typical brittle fracture characteristic with exposed clean fiber bundles. Fracture surfaces of the PC0 and PC0-M laminates are shown in Fig. 12b and c, respectively. The fracture surface of PC0 is relatively smooth and flat (Fig. 12b), and this indicates weak resistance to crack propagation. In contrast, a protruding shape (indicated by the black dashed arrow) on the fracture surface of the laminate interleaved with modified pure PA12 film can be observed in Fig. 12c. It is likely that toughening was caused by good interfacial adhesion between the film and CF/EP, and this is proved by a high magnification image of the rough rupture morphology shown in the inset of Fig. 12c. A phase separation structure can be observed. Plasma treatment can introduce active hydrogen-containing functional groups into the polymer chains on the surface of the film, and this is shown in the FTIR analysis. Active hydrogen-containing functional groups can participate in the curing reaction of the epoxy resin and make it possible to enhance the interfacial adhesion through chemical covalent bonds. This provides an explanation for the improved value of *G*_IC_ for PC0-M compared to that of PC0. Fig. 12d and e show the fracture surfaces of PC10 and PC10-M samples, and there is a distinct difference between these surfaces. For PC10 (Fig. 12d), the fracture surface is flat, and at higher magnification, it can be seen that many MWCNT bundles were pulled out (inset in Fig. 12d). This may be because of the chemically inert surface of the CTF, which leads to poor adhesion with the epoxy. Fig. 12e shows that the fracture surface of the PC10-M is rougher. The vertical fracture surfaces (red solid arrow) indicate that the crack deflects into the interior of the CTF, which might occur at carbon nanotube aggregates; this shows a zigzag crack propagation path. Also, we can observe grooves left from fiber debonding between the CTF and CF/EP in Fig. 12f, and this indicates a good combination. In addition, Fig. S2† shows cross-sections of PC10 and PC10-M in the fiber direction at low magnification. In Fig. S2a,† the interface between the inserted film and the CF/EP phase is clearly visible. However, the interface becomes obscured after plasma treatment (Fig. S2b†), and this is because plasma treatment increased interfacial compatibility. Also, in a higher magnification image (Fig. 12g), it can be observed that a large number of carbon nanotubes were pulled out (indicated by the black dot oval) in the vertical fracture surfaces, and these hindered the growth of cracks *via* a bridging effect. The crack propagates with a large amount of such crack deflections; combined with carbon nanotube bridging, this indicates that a large amount of fracture energy can be dissipated and absorbed.

Fig. 12h shows the interfacial morphology of the CTF and the epoxy matrix, and this provides additional useful evidence for strong interfacial adhesion and confirms the cohesive failure mode. The fracture surface between the CTF and the epoxy matrix exhibits irregular morphology and nanoscale phase separation at the two-phase interface. This may indicate that the surface of the CTF after plasma treatment is activated *via* the introduction of hydrogen-containing functional groups to improve interfacial compatibility. Phase separation formed with the help of the curing dynamics of the epoxy resin.^29^ This nanoscale phase separation structure contributes to the greater energy absorption abilities of the matrix while maintaining its mechanical and thermal properties.^19^ A lot of shear band structures (indicated by the blue dashed arrow) can be observed in a higher magnification image of the fracture surface. In contrast, this phenomenon is not observed in PC0-M (Fig. 12c), and this may be because debonding and pull-out of CNTs promote plastic deformation of the surrounding matrix, dissipating more fracture energy.^55^ All of these observations contribute to the increased interlaminar fracture toughness of the CF/EP laminates interleaved with plasma-treated CTF.

#### 3.4.3 Stress transfer model

**Fig. 13:**
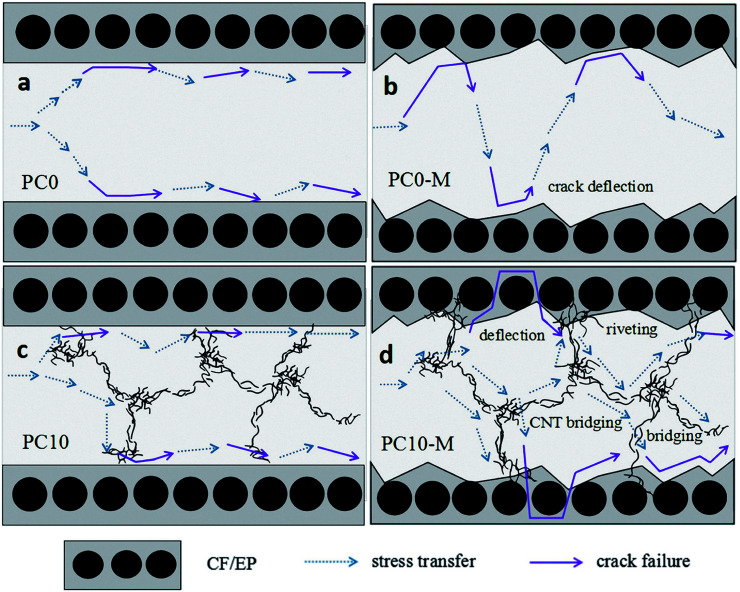
Schematic model of stress transfer in the interlaminar region of composites with different interlayers: (a) PC0, (b) PC0-M, (c) PC10, and (d) PC10-M.

Fig. 13 shows a schematic model of stress transfer in the interlaminar region of the CF/EP composites with different interlayer films. As shown in Fig. 13a, the unmodified pure PA12 film interlayer did not improve toughness, and there was weak resistance against delamination at the interface. Fig. 13b shows the crack propagation behavior of the composites with the modified pure PA12 interlayer film. The presence of chemical bonds between the interfaces led to effective stress transfer from the resin matrix to the PA polymer chains. Ductile deformation of the PA chain dissipates more fracture energy. In addition, cracks are deflected or pinned at the interfacial layer of the plasma-treated pure PA12 film interlayered composite. Fig. 13c shows the crack propagation behavior of the unmodified CTF interlayer composites. Because of poor interfacial adhesion, MWCNT agglomerates are considered to be points of stress concentration that can accelerate crack propagation. Fig. 13d shows the crack propagation behavior of the composites with the modified CTF. Strong interfacial bonding and bridging of MWCNT-EP at the interface result in crack deflection and more effective stress transfer from the resin matrix and carbon fiber to the CTF system. A network of MWCNT aggregates facilitates stress redistribution in the layer, and combined with the bridging effect of the MWCNTs, this network effectively mitigates the advance of the crack front and inhibits propagation of cracks.

## 4. Conclusions

In this work, the effect of MWCNT-doped PA12 film interleaves on the electrical conductivity and interlaminar toughness of CF/EP laminates was systematically investigated. Interleaving the MWCNT-doped film significantly increased electrical conductivity of the CF/EP composite laminates and the conductivity of laminates interleaved with oxygen plasma-treated MWCNT-doped film was significantly improved compared to that without plasma treatment. Experimental results show that electrical conductivities in the *Y* and *Z*-directions of laminates interleaved with 10 wt% MWCNT-doped film after plasma treatment increased 2-fold and 21-fold, respectively, compared to the control sample. This is attributed to the increased dispersibility of CNTs in the resin matrix because of better dissolution of the doped film in the composite system, and this further reduced the resistance of the resin-rich region. Interlaminar toughening of CF/EP composite laminates was also achieved *via* interleaving of plasma-treated, porous MWCNT-doped films. DCB test results demonstrated that Mode-I fracture toughness (*G*_IC_) and resistance (*G*_IR_) of the plasma treated 10 wt% MWCNT-doped film laminates increased by 59.3% and 112.8%, respectively. Enhanced interlaminar toughening was mainly attributed to chemical bonds formed at the interface of the doped system and CF/EP composite; chemical bonding improved interfacial adhesion and resulted in good stress transferability from the brittle epoxy matrix to the ductile PA phase. This combined with CNT pull-out and CNT bridging resulted in tougher laminates. This research is important for simultaneously improving the interlaminar facture toughness, and the LSP and EMI properties of CFRP used in the aerospace industry.

## Conflicts of interest

There are no conflicts to declare.

## Supplementary Material

RA-008-C8RA05366A-s001
