# Supplementary material for: Simultaneous enhancement of electrical conductivity and interlaminar fracture toughness of carbon fiber/epoxy composites using plasma-treated conductive thermoplastic film interleaves
Source: RSC Adv. 2018 Jul 30;8(47):26910–21. doi: 10.1039/c8ra05366a (PMC9083302; doi:10.1039/c8ra05366a)
Supplement: RA-008-C8RA05366A-s001 [file RA-008-C8RA05366A-s001.pdf]

Electronic Supplementary Material (ESI) for RSC Advances.
This journal is © The Royal Society of Chemistry 2018

Supporting Information

Simultaneous enhancement of electrical conductivity and interlaminar fracture toughness of carbon fiber/epoxy composites using plasma-treated conductive thermoplastic film interleaves

Wei Li [1], Dong Xiang [1, *], Lei Wang [1], Eileen Harkin-Jones [2], Chunxia Zhao [1], Bin Wang[1], Yuntao Li [1, *]

W. Li, Dr. D. Xiang, L. Wang, Dr. C. X. Zhao, Prof. B. Wang, Prof. Y. T. Li

1. School of Materials Science and Engineering, Southwest Petroleum University, Chengdu 610500, China

E-mail: dxiang01@hotmail.com;

yuntaoli@swpu.edu.cn

Prof. E. Harkin-Jones

2. School of Engineering, University of Ulster, Jordanstown BT37 0QB, UK

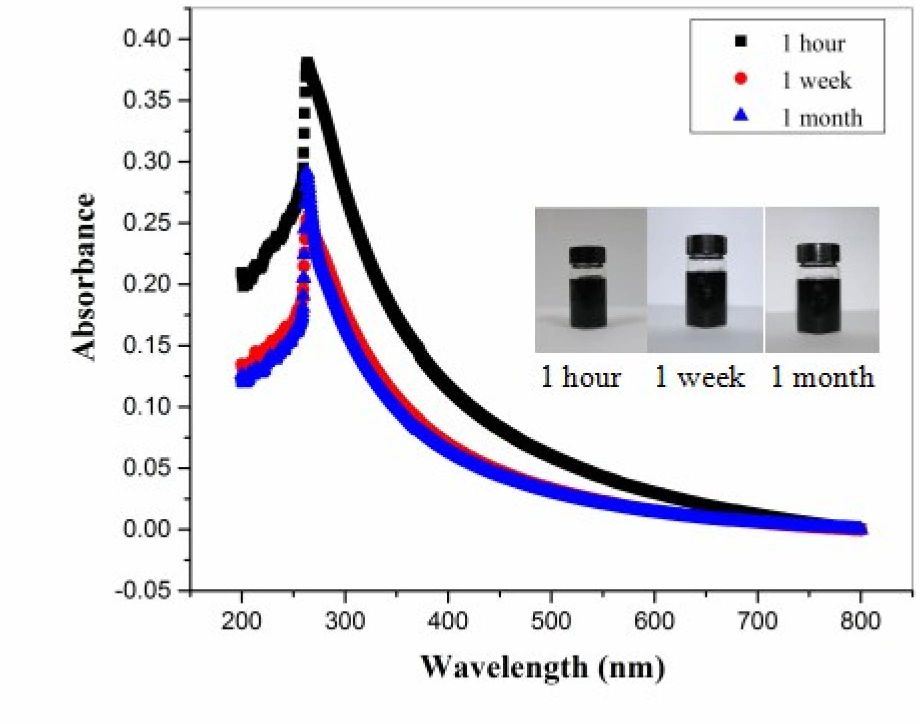

Fig.S1. Typical UV−Vis spectra of 15 wt% MWCNT/DMF suspension. Inset: optical images of diluted suspension after one hour, one week, and one month. The suspension was diluted by 250 times for characterization. UV-Vis measurements were carried out from 200 to 800 nm.

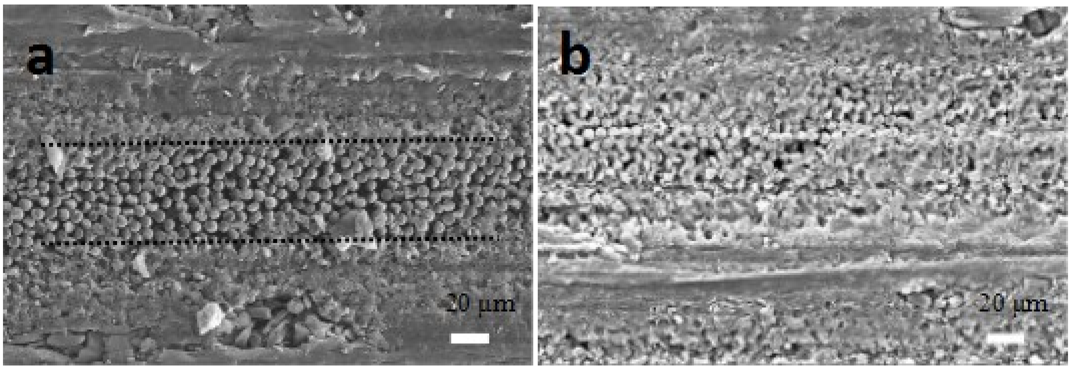

Fig.S2 Cross-sections of (a) PC10 and (b) PC10-M in the fiber direction at low magnification.
